# Optimizing Orthognathic Surgery: Leveraging the Average Skull as a Dynamic Template for Surgical Simulation and Planning in 30 Patient Cases

**DOI:** 10.3390/jcm12247758

**Published:** 2023-12-18

**Authors:** Hsiu-Hsia Lin, Jyun-Cheng Kuo, Lun-Jou Lo, Cheng-Ting Ho

**Affiliations:** 1 Craniofacial Research Center, Chang Gung Memorial Hospital, Taoyuan City 333, Taiwan; sharley@hust.edu.tw; 2 Dental Department of TuCheng Hospital, New Taipei Municipal, New Taipei City 236, Taiwan; popop5541@cgmh.org.tw; 3 Department of Plastic and Reconstructive Surgery, Craniofacial Research Center, Chang Gung Memorial Hospital, Chang Gung University, Taoyuan City 333, Taiwan; lunjoulo@cgmh.org.tw; 4 Division of Craniofacial Orthodontics, Department of Dentistry, Chang Gung Memorial Hospital, Taoyuan City 333, Taiwan

**Keywords:** virtual planning, orthognathic surgery (OGS), average skull template, guiding framework, surgical simulation

## Abstract

Virtual planning has revolutionized orthognathic surgery (OGS), marking a significant advancement in the field. This study aims to showcase the practical application of our established 3D average skull template as a guiding framework for surgical planning, and to share valuable insights from our clinical experience. We enrolled 30 consecutive Taiwanese patients (18 females and 12 males) who underwent two-jaw orthognathic surgery with surgical simulation, utilizing the average skull template for planning. Results indicate the method’s applicability and precision. By adhering to the surgical plan, post-operative outcomes closely aligned with the average skull template, showing negligible deviations of less than 2 mm. Moreover, patients expressed high satisfaction with post-surgery facial changes, with the chin appearance receiving the highest satisfaction scores, while the lowest scores were attributed to nose appearance. Notably, the substantial change in lower jaw position post-mandibular setback surgery contributed to increased satisfaction with the chin position. In conclusion, this study does not seek to replace established surgical planning methods, but underscores that utilizing an average skull as a surgical design template provides a viable, accurate, and efficient option for OGS patients.

## 1. Introduction

The evolution of digital technology and software has led to innovative approaches in orthognathic surgical (OGS) planning, enhancing its precision and efficiency. Traditional two-dimensional (2D) methods involving paper-based procedures and manual crafting have shifted to 3D technology applications, which overcome the limitations of time-consuming 2D methods and enhance detection in radiographs [1]. In the 3D field, simulations with dental casts using cone beam computed tomography (CBCT) and dental impression alternatives like oral scanning have emerged [2,3,4,5]. Despite the popularity of 3D methods, reliance on cephalometric measurements and norm values persists. A recent approach utilizing a three-dimensional average skull template for planning has been validated and applied in our center [6], streamlining the diagnosis of abnormal jaw positions and enabling surgery simulation without model surgery or angular measurements.

Class III malocclusion is prevalent in Asia (3–5% in Japan, around 2% in China) [7], where patients exhibit mandible prognathism, with or without maxillary retrognathism, which impacts their appearance and psychosocial status. Orthognathic surgery (OGS) aims for a balanced facial profile, correct lower jaw positioning, and optimal dental occlusion [8,9]. Our prior research established a 3D cranial model integrated into virtual surgical planning (VSP) for repositioning the maxilla and mandible. For class III patients, OGS seeks to standardize jaw size, position, and aesthetics, which often involves mandibular setback using bilateral sagittal split osteotomy (BSSO) and maxillary advancement through LeFort I osteotomy. However, BSSO poses challenges, including potential inferior alveolar nerve injury.

Our previous study [6] demonstrated discrepancies of less than 1 mm in facial landmarks compared to 3D cephalometric measurements and surgical simulation with a 3D average skull template, validating its accuracy and clinical suitability.

This study aims to apply the average skull template to prospective patients, in order to guide surgical planning and evaluate outcomes and patient satisfaction for clinical dissemination. Future integration of artificial intelligence into surgical planning using the average skull template holds clinical potential [10,11,12]. Refined surgical planning simulations based on the average skull template and validated results will contribute to a machine learning facial classification model. The model will predict surgical plans for upcoming patients, providing initial references for clinical physicians, significantly reducing planning time and enhancing precision based on individual patient conditions and surgical assessments.

## 2. Materials and Methods

**Figure 1:**
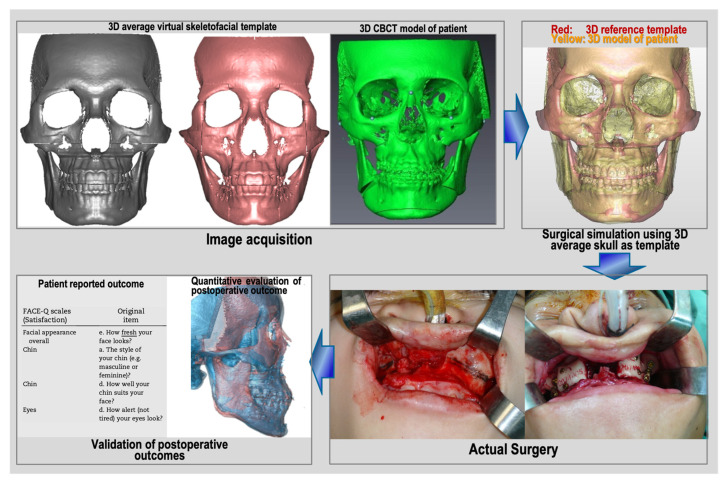
Flowchart of this prospective study’s design.

This study included 30 Taiwanese patients (18 females, 12 males) undergoing two-jaw orthognathic surgery (OGS) with surgical simulation using the average skull template, from August 2021 to July 2022. The patients, aged 22 to 38 years, presented with class III malocclusion, facial asymmetry, concave profile, and other related issues. All of them underwent cone beam computed tomography (CBCT) scans for surgical simulation. The procedures were performed by the same orthodontist and surgeon. Exclusions comprised severe deformities, prior orthognathic surgery, and facial trauma. Institutional Review Board approval was obtained (Chang Gung Medical Foundation IRB 202002305B0), and the participants provided informed consent. The methodology for constructing the 3D craniofacial model and 3D average skull-based surgical planning was outlined in a previous publication [6]. The study’s flowchart (Figure 1) details four stages: 3D image acquisition, surgical simulation, actual surgery, and post-operative outcome validation.

### 2.1. Three-Dimensional Image Acquisition

**Figure 2:**
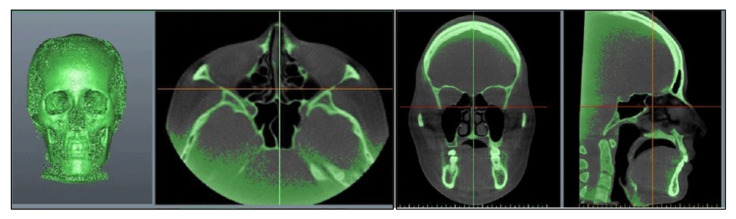
Reconstructing the 3D bone structure by importing 3D CBCT images.

Participants underwent pre-surgical CBCT scans using the KaVo ORTHOPANTOMOGRAPH™ OP 3D Vision X-ray system (DEXIS™, Quakertown, PA, USA) with a low-dose protocol. The scans, performed 2 weeks pre-surgery, used 120 kVp, a voxel size of 0.4 mm × 0.4 mm × 0.4 mm, 26-s scan time, and an 11 cm × 23 cm × 17.3 cm field of view. Head orientation ensured that the Frankfort horizontal plane was parallel to the ground. The patients were instructed to avoid swallowing, to keep their mouths closed, and maintain a centric occlusion bite. The resulting DICOM-format image data, with 0.4 mm slice thickness, were processed in Materialise ProPlan CMF 3.0 (Leuven, Belgium) to reconstruct and analyze 3D bone and soft tissue models (Figure 2). Threshold values of 300–400 HU and 800–900 HU distinguished the hard and soft tissues, respectively. All CBCT data were stored in the Chang Gung Craniofacial Research Center’s database.

### 2.2. Surgical Simulation Using 3D Average Skull as Template

**Figure 3:**
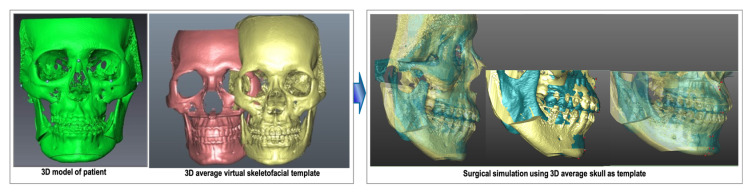
The surgical simulation using 3D average skull as template.

The average skull served as a guide for surgical simulation, assessing deviations between two skull images in millimeters (sagittal, frontal, vertical). Using segmentation, the maxilla and mandible were delineated, and 3D CBCT dental structures were replaced with intra-oral scanner-acquired digital dental images using 3Shape, TRIOS^®^ 3 (3Shape company, Copenhagen, Denmark). Dolphin Imaging^®^ 11.95 software (Chatsworth, CA, United States) on the composite skull model established the virtual surgical occlusion setup, adjusting mandible position for normal overjet, overbite, midline alignment, and arch coordination. The setup considered a 15% surgical relapse with planned overcorrection (3–4 mm incisal overjet) in class III cases. Once the final virtual occlusion was confirmed, the maxilla and mandible were occluded to create the maxillomandibular complex object (MMC). Surgical simulation positioned the MMC close to the average skull template’s maxillary, mandibular, and upper incisal positions (Figure 3) [6]. The three-step simulation process involves importing the 3D model template, identifying landmarks, superimposing the template and patient images, and conducting the simulation based on the MMC’s position and pitch rotation. Detailed procedural descriptions are available in our previous publication [6].

To validate the intra-observer reproducibility of landmark identification, 10 subjects were randomly selected for repeated testing. The defined landmarks were re-located in a 2-week interval by the same investigator (CT Ho). The intra-rator errors were analyzed by calculating the Euclidean distance between first and second landmarks’ coordinates, A1 (X_A1_, Y_A1_, Z_A1_) and A2 (X_A2_, Y_A2_, Z_A2_), respectively, in a 3D coordinate system using the following formula:

$$Distance\left(d\right)=\sqrt{\left[{\left(X_{A1}-X_{A2}\right)}^{2}+{\left(Y_{A1}-Y_{A2}\right)}^{2}+{\left(Z_{A1}-{ZY}_{A2}\right)}^{2}\right]}$$

### 2.3. Actual Surgery

We utilized a modified Hunsuck bilateral sagittal split osteotomy, LeFort I osteotomy, and, when necessary, genioplasty for all patients, following the single-splint two-jaw orthognathic surgery (OGS) method [13]. Full mobilization of the maxilla and mandible was achieved, adjusting the distal mandibular segment using the final occlusal splint to establish occlusion with the maxilla, creating the maxillomandibular complex (MMC). The MMC was repositioned based on the planned position, guided by a 3D average skull template through a customized positioning guide. Two guides were crafted using computer-aided design and manufacturing: one for LeFort I osteotomy, and another for positioning the MMC. The LeFort I guide was precisely placed on the maxilla to guide the osteotomy lines. After LeFort I osteotomy, the segments were secured onto the final occlusal splint. Using the positioning guide, the MMC was accurately relocated, temporarily secured with screws, and the facial skin was redraped for assessment. Lateral maxilla plate fixation followed, aligning the dental midline, skeletal midline, facial skin midline, occlusal plane, upper tooth show, facial proportion, and symmetry. The mandibular ramus fixation utilized percutaneous bicortical screws, with genioplasty if needed. Notably, guides were not used for mandibular ramus osteotomy, fixation, or genioplasty [14].

### 2.4. Validation of Post-Operative Outcomes

#### 2.4.1. Quantitative Evaluation of Post-Operative Outcome

To assess the discrepancy between post-operative results and the average skull, 3D models were converted into stereolithography (STL) files for registration purposes. The average skull models were superimposed on the patients’ skull models in the registration region (orbitale, frontal, the upper third or half of the nose, and external zygoma) based on seven pairs of anatomical landmarks (N, Or (L, R), Lo (L, R), Zy (L, R)), with image resizing performed to achieve the best alignment using the best-fit method in both the anterior and lateral views [14]. Subsequently, positional discrepancies (in millimeters) were evaluated at key anatomical points, including the maxilla (point A), the midcontact point of the upper incisors (U1C), the mandible (point B), and the chin (points Pog and Me). These image discrepancies pertaining to the maxilla, mandible, chin, and upper incisors were visualized and quantified using 3D software (Materialise ProPlan CMF 3.0), utilizing five pairs of anatomical landmarks (A, B, Pog, Me, U1).

#### 2.4.2. Patient-Reported Outcome Questionnaires

Patients completed self-administered questionnaires to evaluate their facial appearance satisfaction six months post-surgery. This timing allowed for reduced facial swelling and enhanced tissue stability. Two measures were employed: overall appearance rating (OAR) and satisfaction with facial appearance (SFA) [14]. The OAR assesses ideal facial appearance perception on a 0 to 100 scale, with 1 being extremely unattractive and 10 extremely attractive. The SFA gauges satisfaction with specific features (nose, cheeks, lips, gum display, teeth, chin, and facial width) on a 1 to 10 scale, where 1 is very dissatisfied and 10 is very satisfied. Higher scores indicate increased satisfaction and facial attractiveness.

### 2.5. Statistical Analysis

The Pearson correlation coefficient was adopted to validate the intra-observer reproducibility. The range is between 0 and 1, with a higher value indicating a higher correlation or reliability. The means and standard deviations of the measurements were obtained for descriptive statistics. The data were verified to be normally distributed using the Kolmogorov–Smirnov test. The paired *t*-test was adopted for statistical comparisons between pre- and post-operative changes on cephalometric analysis. A *p* value of 0.05 was considered statistically significant. Statistical analyses were performed using SPSS Statistics for Windows, Version 17.0 (released 2008, SPSS Inc., Chicago, IL, USA).

## 3. Results

The mean intra-observer difference in landmark identification was 0.37 mm (range: 0.31–0.49 mm), and the Pearson correlation coefficients (*r* = 0.88–0.98; all *p* < 0.05) revealed significant correlations between the investigators’ observations, indicating that the virtual-guided data collection was accurately and consistently performed (Table 1).

Table 2 presents the differences between post-operative results and the average skull. The mean differences were computed in three anatomical planes: the transverse plane (*x*-axis, mediolateral), the sagittal plane (*y*-axis, anteroposterior), and the vertical plane (*z*-axis, superiorinferior). The study did not include patients with positional differences exceeding 3 mm in any direction. All of the data points exhibited deviations of less than 2 mm, except for Pog in the anteroposterior direction (2.3 mm). U1 mid in the *y*-axis displayed a measurement discrepancy of 1.9 mm, and Me in the vertical direction showed a deviation of 1.9 mm, which closely approached the 2 mm threshold. This suggests a tendency toward greater alignment in the *x*-axis for all landmarks compared to the y- and z-axes, as measurements in the *x*-axis were smaller than those in the other two axes. Furthermore, there was less deviation observed in the maxilla (point A) than in the mandible (points B and Pog) in all three axes. Notably, the maxilla (A point, x = 0.9, y = 1.1, z = 1.0) exhibited the highest degree of alignment among all landmarks. This study introduces a novel approach, and as of now, substantial results are not directly available. Following the “rule of thumb” established in previous studies, we conducted initial testing with a sample size of 30 [15]. A sample size of 30 is sufficient for a post hoc power analysis of 0.75 to detect the difference.

The overall appearance rating (OAR) and satisfaction with facial appearance (SFA) results are shown in Table 3. The highest level of satisfaction was observed in terms of chin appearance, while the lowest score was recorded for nose appearance. All of the scores were close to 9, indicating a high level of satisfaction. A significant alteration in the position of the lower jaw following mandibular setback may contribute to a higher satisfaction score for chin position.

## 4. Discussion

Leveraging 3D simulation techniques provides a distinct advantage in refining surgical planning precision by adjusting the yaw, roll, and pitch rotation of the osteotomized bony segment. Additionally, the 3D simulation detects and addresses collisions or gaps between bony segments, leading to improved surgical outcomes. Moreover, it expedites treatment planning and proves to be a more cost-effective alternative compared to traditional methods [16,17,18].

To our knowledge, this marks the first assessment of treatment outcomes in patients with class III malocclusion and facial asymmetry undergoing two-jaw surgery, utilizing a 3D average skull as a reference template for surgical planning. The study has limitations in terms of its generalizability, as the sample for the average skull template comprises only normal subjects from Taiwan, making it primarily applicable to Chinese or Asian populations. However, the methodology can be extended to address malocclusions or facial deformities more broadly. Another limitation is the static approach of the study, as it focused on hard tissue surgical simulation using an average template, which makes it challenging to accurately predict soft tissue changes and dynamics, such as gum exposure during smiling. Videos could serve as a valuable tool for dynamically assessing and recording soft tissue, necessitating a final adjustment to the surgical plan after clinical evaluation. Therefore, final modifications are still needed after clinical evaluation for dynamic soft tissue expression.

Accurately predicting post-operative facial appearance during surgical planning simulation requires a comprehensive understanding of the relationship between the displacement of soft tissues and bone tissues (hard tissues) before and after surgery. This understanding, which is not limited to specific regions, necessitates a holistic approach (non-localized analysis) that acknowledges that the proportion is not one-to-one. To address this, we plan to utilize deep learning techniques to establish a three-dimensional orthognathic surgery soft tissue prediction simulation system. Facial regions impacted by surgery will be subdivided into smaller areas, each with its pre- and post-operative skeletal displacement values (input) and soft tissue displacement values (output). We aim to leverage artificial intelligence (AI) technology, specifically convolutional neural network (CNN), to build a facial soft tissue prediction model. Clinically, this approach enables physicians not only to adjust surgical plans (skeletal displacement) based on predicted facial outcomes, but also to provide a realistic preview of post-surgery facial appearance, acting as a communication tool for easy patient understanding.

Using an average skull as a surgical design model does not aim to replace the current method, but provides a valuable alternative. Compared to existing surgical simulation protocols, our method stands out. Unlike other approaches that require 3D cephalometric analysis involving angular and linear measurements and reliance on norm values to plan osteotomized bony segments, our study is significantly more efficient. We eliminate the need for cephalometric analysis and dental models. By superimposing two skull images, we visualize and calculate deviations from the ideal position in linear measurements across three coordinates. This streamlines the process and enhances communication and education with patients. However, it is important to note that our approach is static, focusing on the average skull model without considering the dynamics of soft tissues.

In our investigation, we utilized Dolphin imaging 11.95 to integrate intra-oral scans with CBCT scans for dental fusion. The accuracy of this fusion process, assessed using commercially available software, demonstrated a high level of precision compared to the established gold standard. Although there may be some inaccuracies in the cranial/caudal directions for both the maxilla and mandible, we addressed this by employing fiducial markers and the best-fit method to refine the fusion process [19]. Nonetheless, a visual check is recommended. To further enhance accuracy in aligning intra-oral scans with dental surfaces in CBCT, we employed fiducial markers and the best-fit method in fusing dental elements from intra-oral scans. Again, a visual check is advised for validation [20].

In this study, we compared positional disparities between the post-operative skull and the average skull using paired landmarks, and administered patient satisfaction questionnaires concerning overall appearance and specific facial regions. These questionnaires, which are validated instruments for patients following orthognathic surgery, assessed perception of appearance with two measures: overall appearance rating (OAR) and satisfaction with facial appearance (SFA) [14]. Minimal jaw bone deviations were observed between paired landmarks, and the patient-reported satisfaction levels were notably high. The success of orthognathic surgery relies on precise surgical planning and patient contentment. This innovative approach presents an alternative tool for virtual 3D surgical planning of orthognathic surgery.

Based on the results in Table 2, three-dimensional analysis revealed minimal discrepancies between the post-operative skull and the average skull template for specific paired landmarks, including point A (maxilla), U1, point B (mandible), Pog, and Me. Most of these paired landmark deviations were small, measuring less than 2 mm in absolute value, except for Pog, which exhibited a 2.3 mm deviation in the anteroposterior direction. Previous studies recommended a 2 mm range as the most suitable criterion for assessing linear differences between planned and actual post-operative images [21,22,23]. Our results fell within this recommended range. The slight anterior shift of Pog by 0.3 mm beyond this range could be attributed to mild relapse in the lower jaw six months post-surgery, influenced by muscular actions on bony tissue, condylar changes, or other contributing factors [24]. The second-largest deviation was observed in the vertical difference at Me (1.9 mm, still less than 2 mm), possibly explained by the facial patterns of selected patients. The facial index range for mesocephalic patterns typically falls within 0.85–0.89 (N-Me/Zy-Zy). Some patients with longer or shorter faces within this range may influence the average data, but the deviations still fall within the 2 mm margin criteria. Regarding U1 mid, exhibiting a 1.9 mm deviation in the *y*-axis, this can be attributed to the modified surgical approach in our protocol. Minimal dental decompensation was performed before surgery, and any remaining discrepancies can be readily corrected through post-surgical orthodontic treatment. Hsu et al. demonstrated that the precision of surgical simulation in orthognathic surgery can result in deviations ranging from 0.6 to 3.5 mm from the virtual plan [25]. Therefore, our study, utilizing this novel method, can aid in presurgical planning and maintain accuracy in orthognathic surgery.

This study demonstrates a higher level of congruence when comparing the deviation of the actual post-operative results to the average skull template in the maxilla (point A, x = 0.9 mm, y = 1.1 mm, z = 1.0 mm) compared to the mandible (point B, x = 1.3 mm, y = 1.2 mm, z = 1.2 mm, and Me, x = 1.6 mm, y = 1.6 mm, z = 1.9 mm) in three-dimensional space. It can be hypothesized that the mandible is occluded with the maxilla, and the chin is distant from the rotation center (maxilla), resulting in less conformity in the lower jaw position due to the longer radius effect from the rotation center. In other words, a smaller deviation in the maxilla may lead to a larger deviation in the mandible. In this study, the highest degree of conformity was observed in the mediolateral direction in all landmarks compared to the y- and z-axes, consistent with our previous reports, where more favorable outcomes were noted in frontal symmetry (*x*-axis, mediolateral direction) in midsagittal plane landmarks when comparing the 3D planning group to the 2D planning group. This finding aligns with the report by Alex Wilson et al. [23]. However, our study observed clinically insignificant deviations in the vertical height of the maxilla (A point 1.0 mm in the *z*-axis). This result contradicts the findings of Alex Wilson et al., who reported the greatest nonconformity in the vertical plane (points A and B). We speculate that the routine use of a positional guide to guide the maxilla into the planned position during surgery in our center may contribute to less error in locating the maxilla [14]. In contrast, Alex et al. used vertical measurements from an external reference point, using the medial canthus to fixed dental landmarks without a bone-to-bone guide, which could result in larger deviations in the vertical direction. We observed an incongruence in the sagittal positioning of the chin (Pog), consistent with Alex Wilson et al., and this discrepancy could be attributed to changes in condylar position or relapse.

To address patient preferences and evaluate post-surgery satisfaction, we administered a patient-reported outcome questionnaire [25,26,27]. According to Table 3, patients exhibited high satisfaction with their overall appearance (scores near 90) when utilizing the average skull template for surgical planning. This heightened satisfaction may positively impact their self-confidence and social interactions. Concerning specific facial areas, the nose received the lowest score (8.3). Some patients expressed worries about nostril base enlargement or a flattened nasal bridge following Le Fort I osteotomy. Changes of this nature could be attributed to the detachment of subnasal soft tissues and muscles during the osteotomy. Main V et al. reported a mean increase in alar base width of 1.176 mm in Le Fort I osteotomy patients [28]. Trevisiol et al. demonstrated an increase in inter-alar width of 1.7 mm in patients who underwent subspinal Le Fort I osteotomy [29]. Mitigating strategies, such as an alar base cinch suture or rhinoplasty, may help address these undesirable nasal aesthetic changes [30]. Some patients expressed dissatisfaction with a broader facial appearance in the frontal view following BSSO setback (score 8.6). This phenomenon is thought to result from an increase in intergonial width and bone overlap in the proximal and distal segments during mandibular setback [31,32,33,34]. Yoshioka et al. reported a 0.45 mm increase in intergonial width in the SSRO group [33]. Choi et al. reported a 2.1 mm increase in intergonial width after SSRO [34]. Chen et al. noted a nonsignificant increase of 1.2 mm in intergonial widths with SSRO setback [32]. However, this phenomenon appeared to normalize and become insignificant over time through bone remodeling [34].

In comparison to Hsu’s study on the accuracy of the CASS protocol for orthognathic surgery, our results showed similarities in the range of positional differences between virtual plans and actual results (0.97–2.35 mm vs. 0.6–3.5 mm), meeting the accepted clinical threshold of 2 mm. However, their method involves collecting anthropometric measurements, stone models, and a patient-specific bite jig, facebow, orientation sensor, and CT scan before surgical simulation, which makes this approach more time-consuming and complex. In our study, we used intra-oral scans to obtain digital dental images for fusing dentition from intra-oral scans in CBCT scans. When the patient’s skull was superimposed on the 3D average template, deviations could easily be visualized and calculated without the need for extensive anthropometric measurements and stone models. Comparing the overall appearance scores in this study to our previous study, which used 3D cephalometric norms as a guideline for surgical planning, both methods yielded similar satisfaction scores (89.6 ± 7.6 vs. 89.7 ± 4.5). Furthermore, these scores surpassed those reported by Liao et al. (89.6 ± 7.6 vs. 82.0 ± 11.8) in their study, where they employed a surgical-first approach for orthognathic surgery [18]. This suggests that our new method is as effective as the currently established approach, but offers the advantages of simplicity, time efficiency, and enhanced surgical planning effectiveness.

While the average skull template proves to be a valuable tool, the ultimate decision still relies on soft tissue considerations, such as ex paranasal depression, incisor exposure, and soft tissue chin thickness, as the 3D template method is static rather than dynamic. Experienced doctors may discern potential surgical plans upon initial clinical evaluation. However, with the aid of this template, they can easily validate their diagnoses, particularly in borderline cases. For less experienced practitioners, this template can serve as a starting point for learning how to accurately diagnose maxillary retrusion, mandibular prognathism, or a combination of both in class III malocclusion cases. Therefore, it proves to be a valuable tool for both training purposes and clinical application.

There has been an evolution in virtual planning for orthognathic surgery (OGS), transitioning from 2D methods to 3D methods, with potential future applications involving artificial intelligence (AI). Our previous preliminary study, which utilized an average 3D skull template as a reference for surgical planning to reposition the maxilla and mandible, demonstrated a high level of consistency with simulation images generated using 3D cephalometric normative data, particularly in terms of jawbone positioning [6]. We believe that long-term follow-up (2–3 years later) would better evaluate its stability for OGS. In our study, we propose a new method of surgical simulation, and the planned jaw bone position using our method is similar to other VSP (virtual surgical planning). However, overcorrection is needed to compensate for possible surgical relapse in severe class III malocclusion. The objective of this study is to assess post-operative outcomes and patient satisfaction with this novel surgical planning method, recognizing that patient-reported outcomes hold great value in evaluating the practicality of new techniques. The results indicate that all post-operative data closely align with the average skull template, and patients express high levels of satisfaction, underscoring the reliability of this method for surgical planning. This approach is straightforward and efficient for diagnosis and the establishment of surgical plans.

## 5. Conclusions

This research offers a precise and efficient alternative for OGS patients, aiding communication with patients and educating young doctors. Future possibilities include integrating AI technology to generate diagnoses and surgical plans swiftly by inputting a patient’s 3D image into the software.

## Figures and Tables

**Table 1:** Intra-observer reproducibility of landmark identification in the 3D coordinate system.

Landmark Definition	Mean Difference (mm)	*r*	*p*-Value
Nasion (N)	0.33	0.96	0.008 *
Lateral orbitale (Lo)	0.31	0.98	0.003 *
External Zygoma (Zy)	0.44	0.88	0.002 *
Orbitale (Or)	0.41	0.94	0.002 *
Anterior nasal spine (ANS)	0.40	0.91	0.006 *
A point (A)	0.34	0.93	0.008 *
B point (B)	0.35	0.89	0.005 *
Pogonion (Pog)	0.37	0.93	0.006 *
Menton (Me)	0.41	0.88	0.006 *
Gonion (Go)	0.49	0.88	0.006 *
U1 incisal tip (U1T)	0.31	0.99	0.003 *
U6 cusp (UR6C, UL6C)	0.37	0.98	0.006 *
L1 incisal tip (L1T)	0.34	0.93	0.005 *
L6 cusp (LR6C LL6C)	0.42	0.92	0.003 *
Mean ± SD	0.37 ± 0.067		

*r*, Pearson correlation coefficient; * *p* < 0.05.

**Table 2:** Differences between images obtained using 3D cephalometric normative data and average 3D skeletofacial model for male participants.

Parameters	Mediolateral	Anteroposterior	Superoinferior	*p*-Value
A point	0.975 ± 0.805	1.150 ± 1.008	1.088 ± 0.686	0.885
U1 mid	1.066 ± 0.704	1.940 ± 2.082	1.216 ± 0.883	0.172
B point	1.379 ± 1.259	1.205 ± 0.570	1.276 ± 0.832	0.908
Pog	1.475 ± 1.338	2.357 ± 0.930	1.395 ± 1.397	0.148
Me	1.627 ± 1.376	1.672 ± 1.285	1.925 ± 1.341	0.854

Data are in millimeters and are presented as mean ± standard deviation. A sample size of 30 is sufficient for a post hoc power analysis of 0.75 to detect the difference.

**Table 3:** The overall appearance rating (OAR) and satisfaction with facial appearance of patient-reported outcomes.

Scale	Orthognathic Surgery-Treated Patients		
	Total	Male	Female
	(*n* = 30)	(*n* = 18)	(*n* = 12)
Overall appearance rating (0–100)	89.6 ± 7.6	88.3 ± 8.7	89.8 ± 7.2
**Facial area satisfaction (0–10)**			
Cheek fullness	8.7 ± 0.9	8.8 ± 1.0	8.5 ± 0.7
Chin	9.1 ± 0.9	9.1 ± 1.0	9.1 ± 0.9
Nose	8.3 ± 1.1 *	8.4 ± 1.1 *	8.2 ± 1.2
Lip	8.9 ± 1.1	8.6 ± 1.3	9.3 ± 0.4
Gum show	8.9 ± 0.9	8.9 ± 1.1	8.9 ± 0.7
Dental alignment	9.0 ± 0.9	8.9 ± 0.9	9.2 ± 0.8
facial width change	8.6 ± 0.8	8.5 ± 0.6	8.6 ± 0.8

Data presented as mean ± standard deviation. Higher scores indicate higher satisfaction. * represents the item with the lowest satisfaction.

## Data Availability

The data presented in this study is available upon request to the corresponding author. As the data belongs to the hospital, it is not publicly accessible.
